# Supplementary material for: Structure analysis suggests Ess1 isomerizes the carboxy-terminal domain of RNA polymerase II via a bivalent anchoring mechanism
Source: Commun Biol. 2021 Mar 25;4:398. doi: 10.1038/s42003-021-01906-8 (PMC7994582; doi:10.1038/s42003-021-01906-8)
Supplement: Supplementary file 2 — Supplementary Information [file 42003_2021_1906_MOESM2_ESM.pdf]

Supplementary Figures and Tables

Structure analysis suggests Ess1 isomerizes the carboxy-terminal domain of RNA polymerase II via a bivalent anchoring mechanism

Kevin E. W. Namitz[1,2,5], Tongyin Zheng[3,5], Ashley J. Canning[1], Nilda L. Alicea-Velazquez[1,4], Carlos A. Castañeda[3]*, Michael S. Cosgrove[1]*, and Steven D. Hanes[1]*

1 Dept. of Biochemistry and Molecular Biology, SUNY-Upstate Medical University, Syracuse, NY, USA 13210

2 Current address, Dept. of Chemistry, Pennsylvania State University, University Park, PA, USA 16802

3 Depts. of Biology and Chemistry, Syracuse University, Syracuse, NY, USA 13244

4 Current address, Dept. of Chemistry and Biochemistry, Central Connecticut State University, New Britain, CT, USA 06050

5 These authors contributed equally

* Corresponding authors

e-mail addresses: haness@upstate.edu, cosgrovm@upstate.edu, cacastan@syr.edu

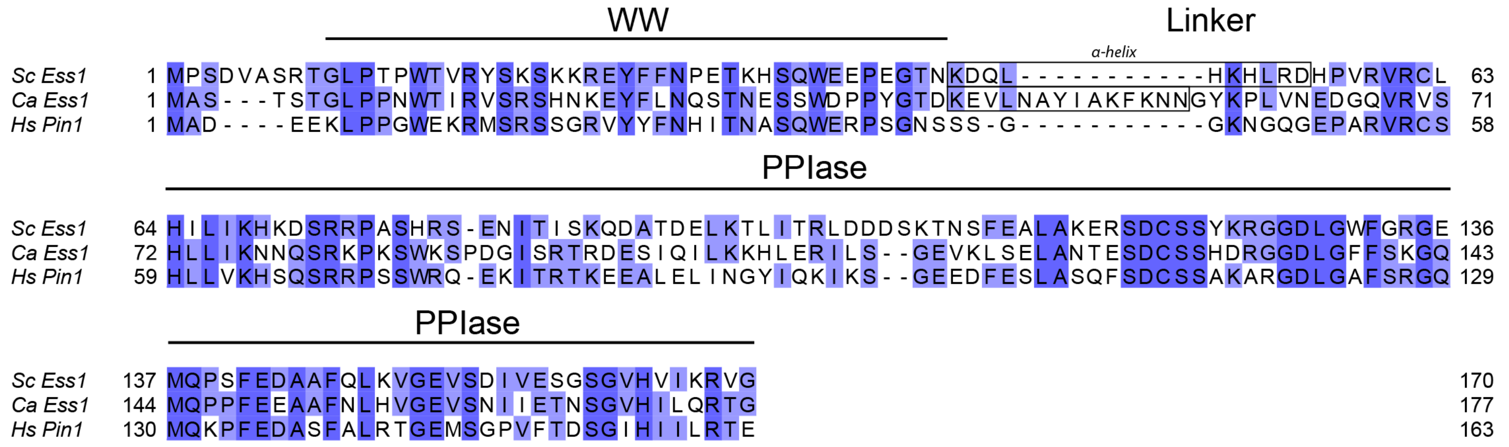

Fig. S1. Sequence alignments. Protein sequences of *S. cerevisiae* Ess1 (Uniprot ID P22696), *C. albicans* Ess1 (Uniprot ID Q59KZ2) and *H. sapiens* Pin1 (Uniprot ID Q13526). The WW, linker and PPIase (catalytic) domains are indicated. Note the sequences of the structured linkers in the fungal enzymes *vs.* the unstructured linker in the human protein. The sequence alignment was performed using T-Coffee (DOI: 10.1006/jmbi.2000.4042) and visualized with Jalview (DOI: 10.1093/bioinformatics/btp033), and residues colored according to % identity.

Fig. S2 – Namitz, Zheng *et al.*

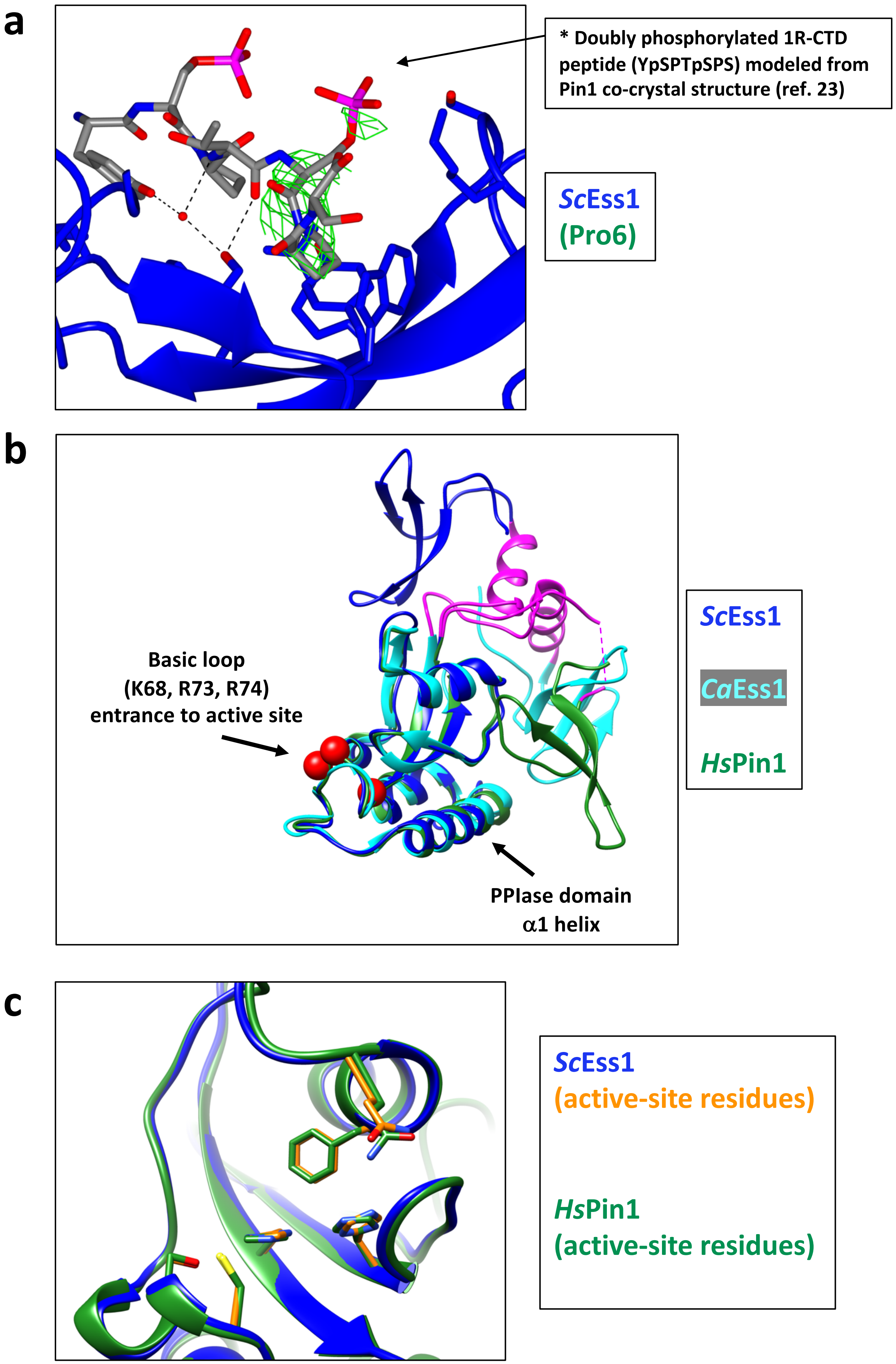

Fig. S2. (a) Simulated annealing composite omit map shows evidence of CTD peptide bound to WW domain in crystal structure. The map (in green - contoured at 2 σ) is only observed over the predicted position of Proline 6 of the CTD peptide seen in a Pin1 cocrystal structure (shown in gray from PDB ID: 1F8A; Verdecia, ref. 23). That peptide was a single heptad CTD repeat phosphorylated at positions Ser2 and Ser5 and bound to the WW of Pin1. It is modeled here to show the position of a putative Pro6 that corresponds to a density we observed in our Ess1 cocrystal.

(b) Overlay of the active site residues from ScEss1 and HsPin1 and HsPin1. ScEss1 (blue protein; orange sidechains) and HsPin1 (green protein; green sidechains) active site residues are absolutely conserved, as is the overall three-dimensional structure of the PPIase active site.

(c) Superposition of the ScEss1, CaEss1 and HsPin1 PPIase domains. The similar folds of these domains are shown, as well as the alterations in the position of the WW domain in comparison to the PPIase domain between each structure. Linker regions joining the WW and PPIase domains are highlighted in pink. Also shown are the conserved residues of the “Basic Loop” (red spheres on Cα of ScEss1 K68, R73 and R74), which enclose the entrance to the active site. Alpha-helix 1 (α1) of the PPIase domain for each structure is also indicated.

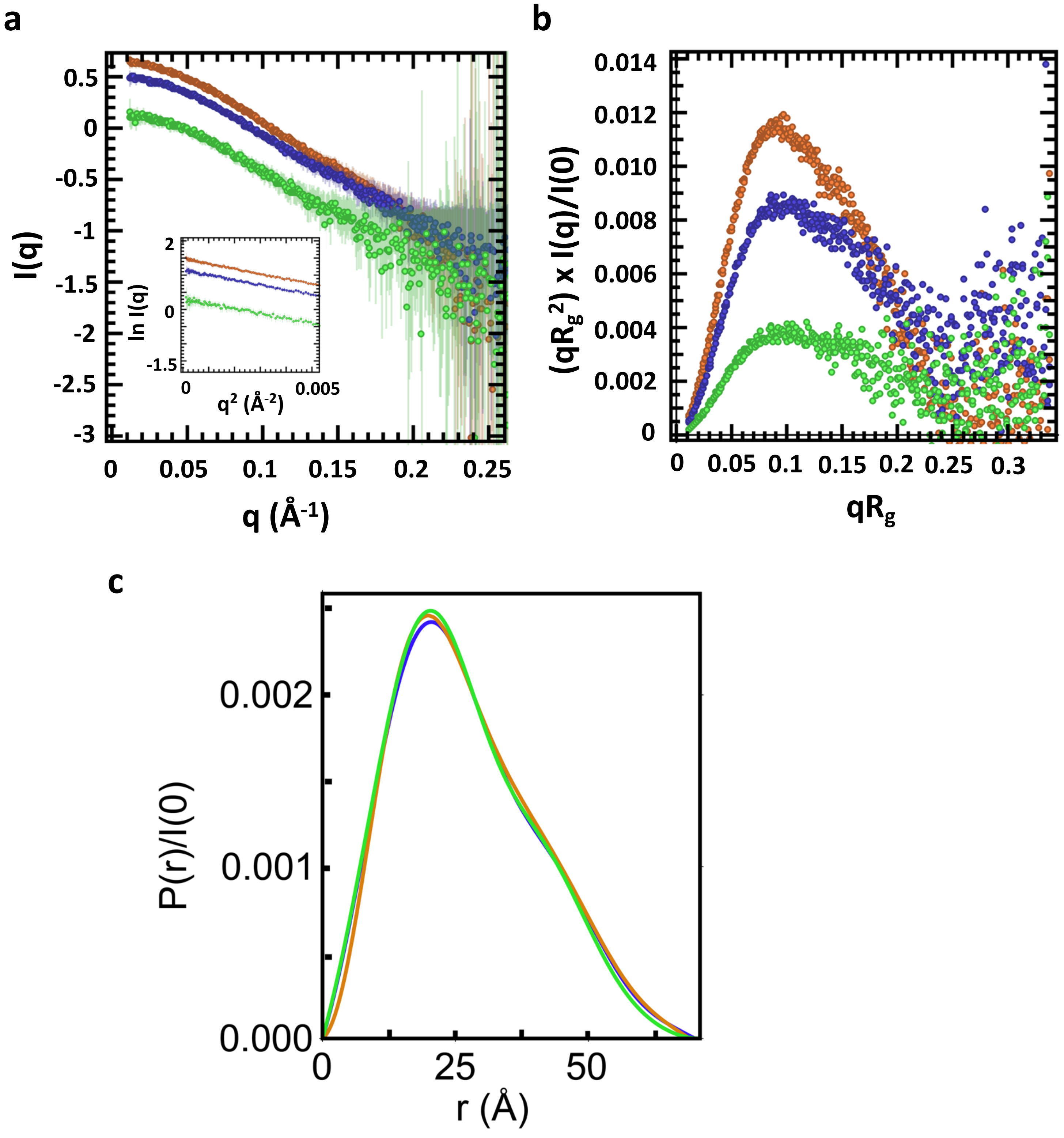

Fig. S3. Biological X-ray scattering data. (**a**) I(q) vs. q plot for 4.78 mg/mL (brown), 3.19 mg/mL (blue) and 1.59 mg/mL (green) ScEss1. Inset: ln I(q) vs. q^2 (Guinier) plot shows no evidence of curvature at low q-angles. (**b**) Dimensionless Kratky plots for the three ScEss1 concentrations show bell-shaped curves, indicating that the proteins are folded. (**c**) P(r) vs. r pairwise distance distributions for each SAXS sample shows a similar d_{max} of 60 - 62Å for each concentration and a similarly-shaped curve, suggesting no changes in oligomeric state over the concentration series.

a

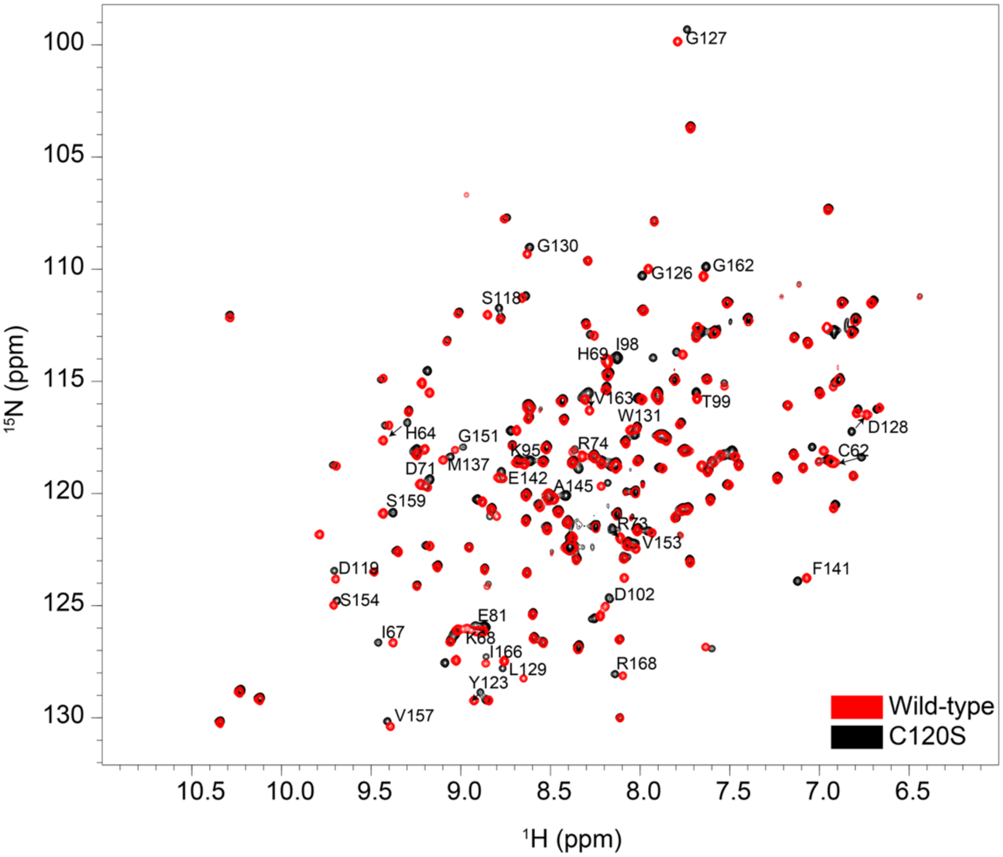

b

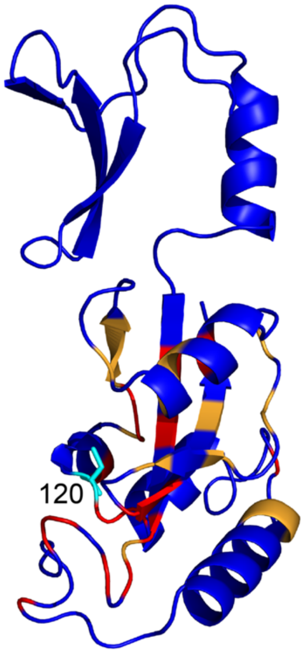

Fig. S4. Wild-type and C120S scEss1 ^{1}H-^{15}N HSQC spectra. (**a**) Wild-type amide chemical shift assignments were visually transferred from C120S spectra. The majority of resonances superpose between the WT and C120S spectra, allowing direct transfer of assignments. The remaining 34 residues (22% of total assigned and marked with residue number) were transferred visually. (**b**) Residues that required visual assignment transfer were mapped on Ess1 structure and colored in orange (CSPs < 0.067 ppm), and red (CSPs between 0.067 and 0.215 ppm). Residue 120 is colored cyan.

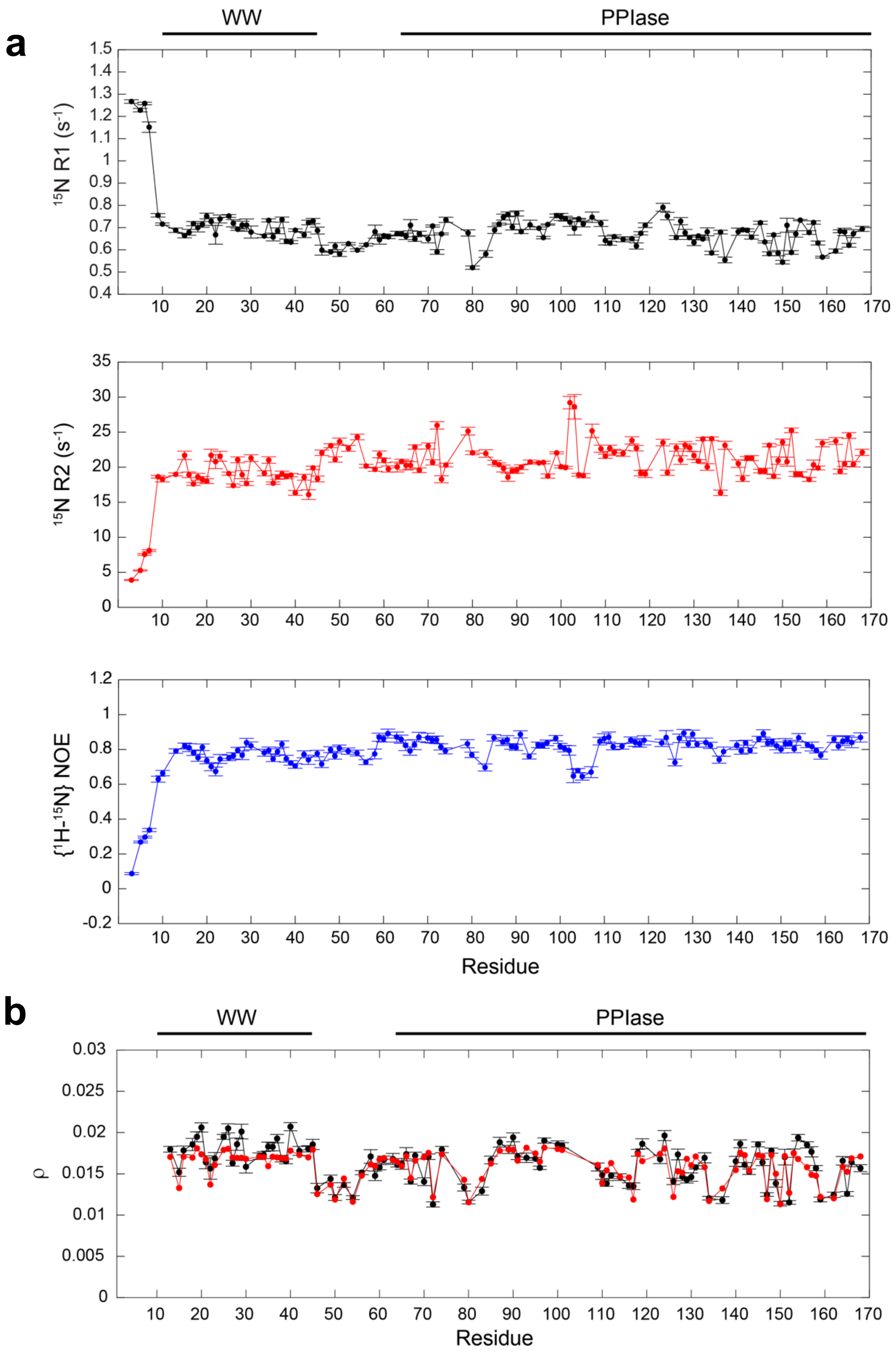

Fig. S5. Backbone ^{15}N relaxation data of Ess1 shows that the protein tumbles as a single unit. (**a**) ^{15}N R_1, ^{15}N R_2, $\{^1H\}$-^{15}N hetNOE for backbone amides, plotted as a function of residue numbers. (**b**) ^{15}N ρ values, plotted as a function of residue number, represent the experimental data (black) and back-calculated values (red) using a fully anisotropic diffusion tensor (see Table S1). Error bars for R_1 and R_2 rates are standard errors of the mean from 500 Monte Carlo trials. Errors in hetNOE and ρ values were determined using standard error propagation.

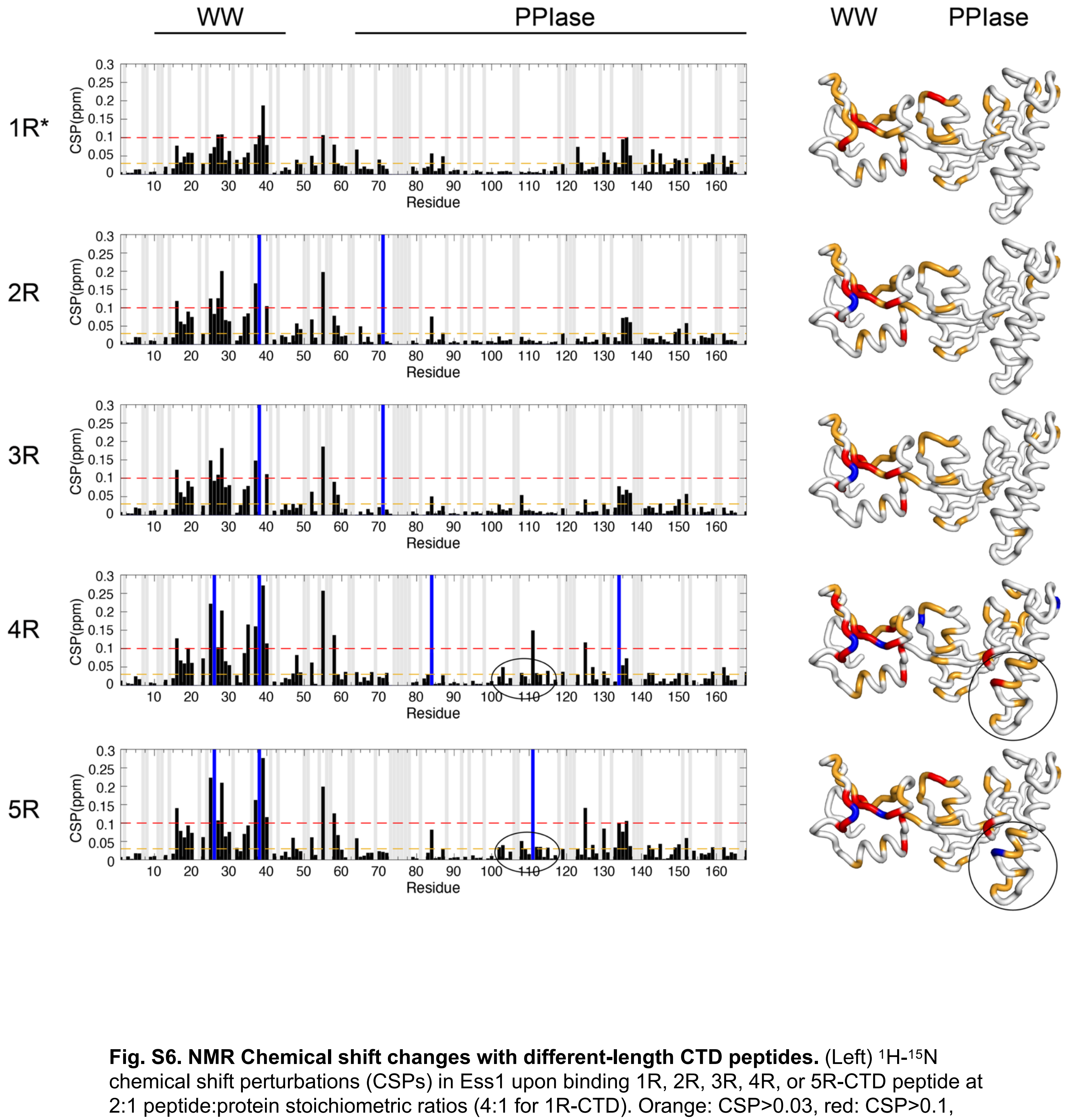

Fig. S6. NMR Chemical shift changes with different-length CTD peptides. (Left) ^{1}H-^{15}N chemical shift perturbations (CSPs) in Ess1 upon binding 1R, 2R, 3R, 4R, or 5R-CTD peptide at 2:1 peptide:protein stoichiometric ratios (4:1 for 1R-CTD). Orange: CSP>0.03, red: CSP>0.1, blue: peak broadened beyond detection. (Right) CSPs are mapped onto the structure of Ess1, and color-coded accordingly.

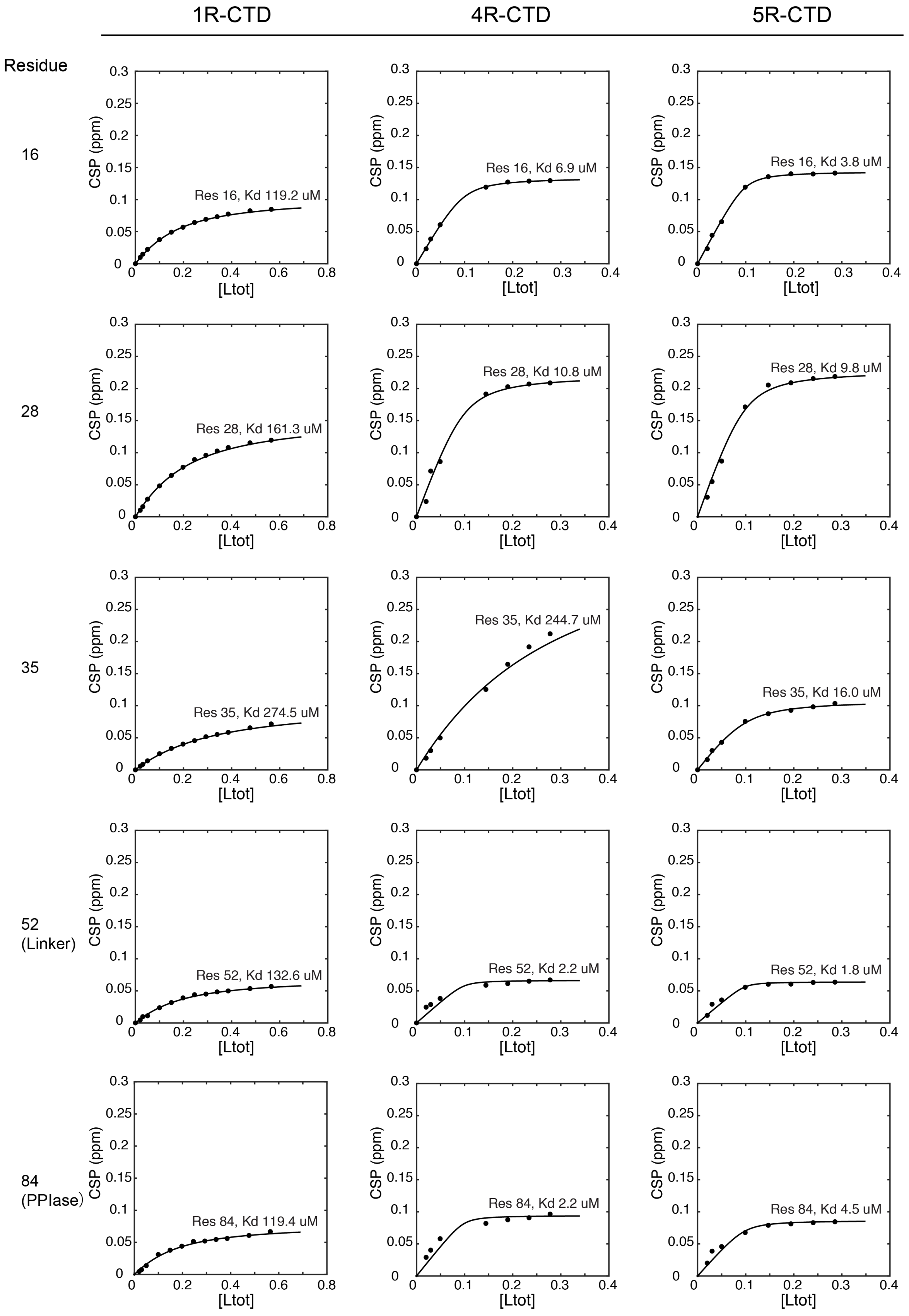

Fig. S7. Representative titration curves of Ess1 residues interacting with CTD peptides of different lengths. A representative selection of Ess1 NMR titration curves used for K_d determination is shown, plotted as CSP vs. ligand:protein ratio. The best fit, using a single-site binding model, is shown as a black line (see Methods).

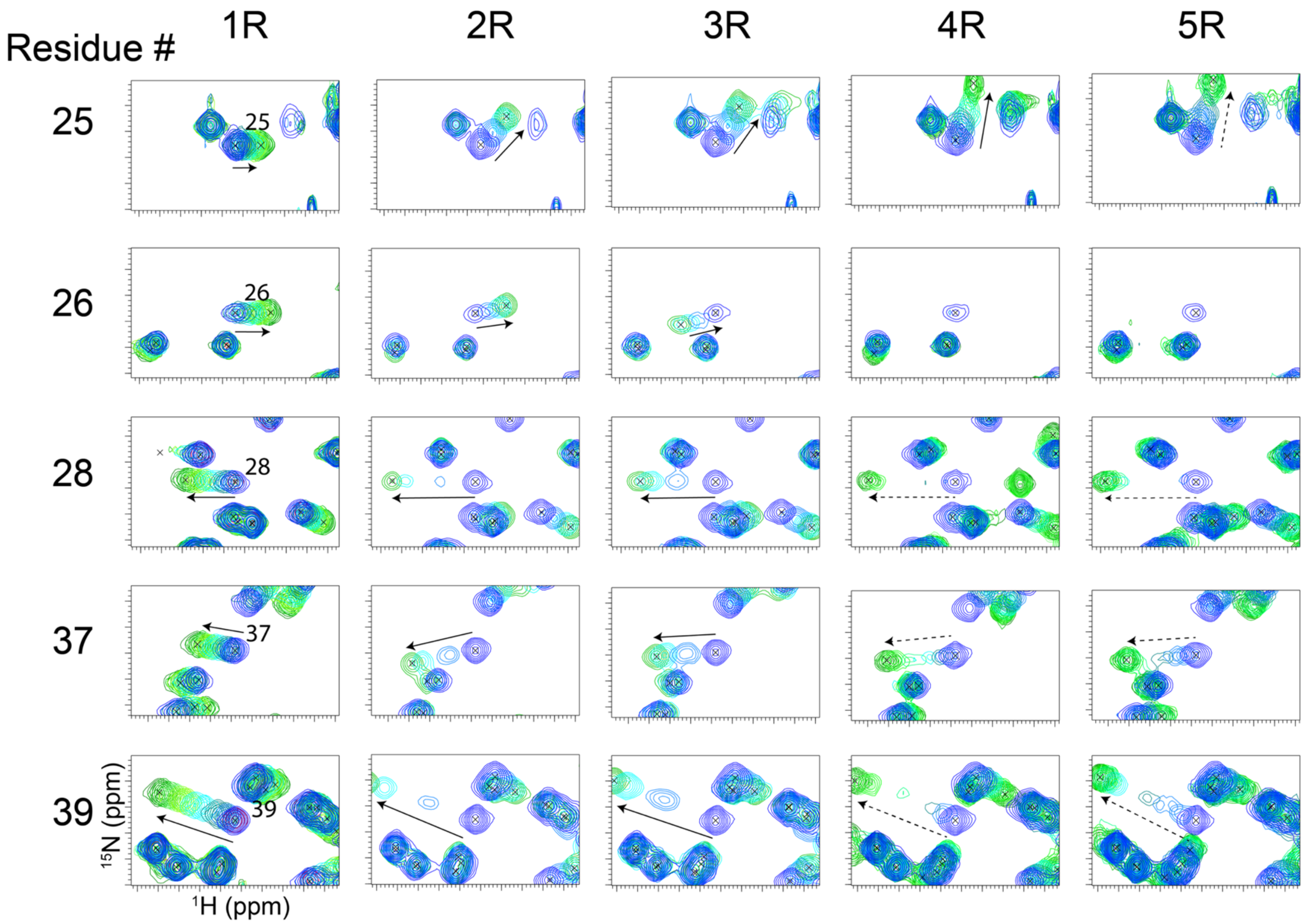

Fig. S8. Peak broadening observed for WW domain residues in Ess1 when interacting with CTD peptides. ^{15}N-^{1}H HSQC spectra of several residues in the WW domain highlight peak broadening during the course of titration experiments with 4R and 5R-CTD peptides; peak broadening was generally not observed for 1R, 2R, and 3R-CTD peptide. NMR spectra at the start and end of the titration are colored blue and green, respectively. Arrows denote amide peak trajectory as peptide ligand was titrated. Contour settings were kept identical across all NMR spectra.

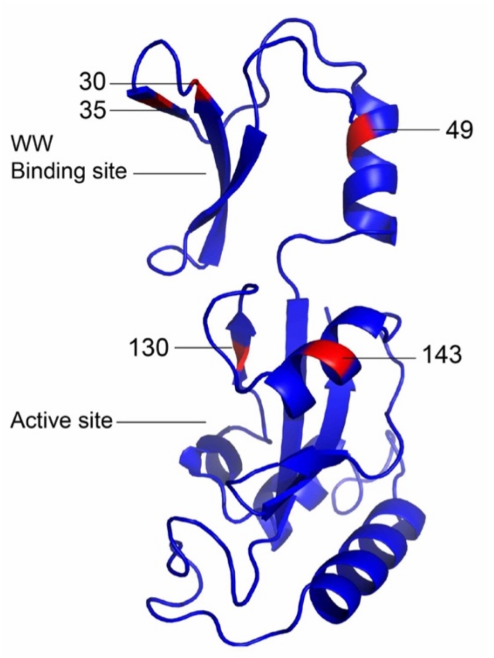

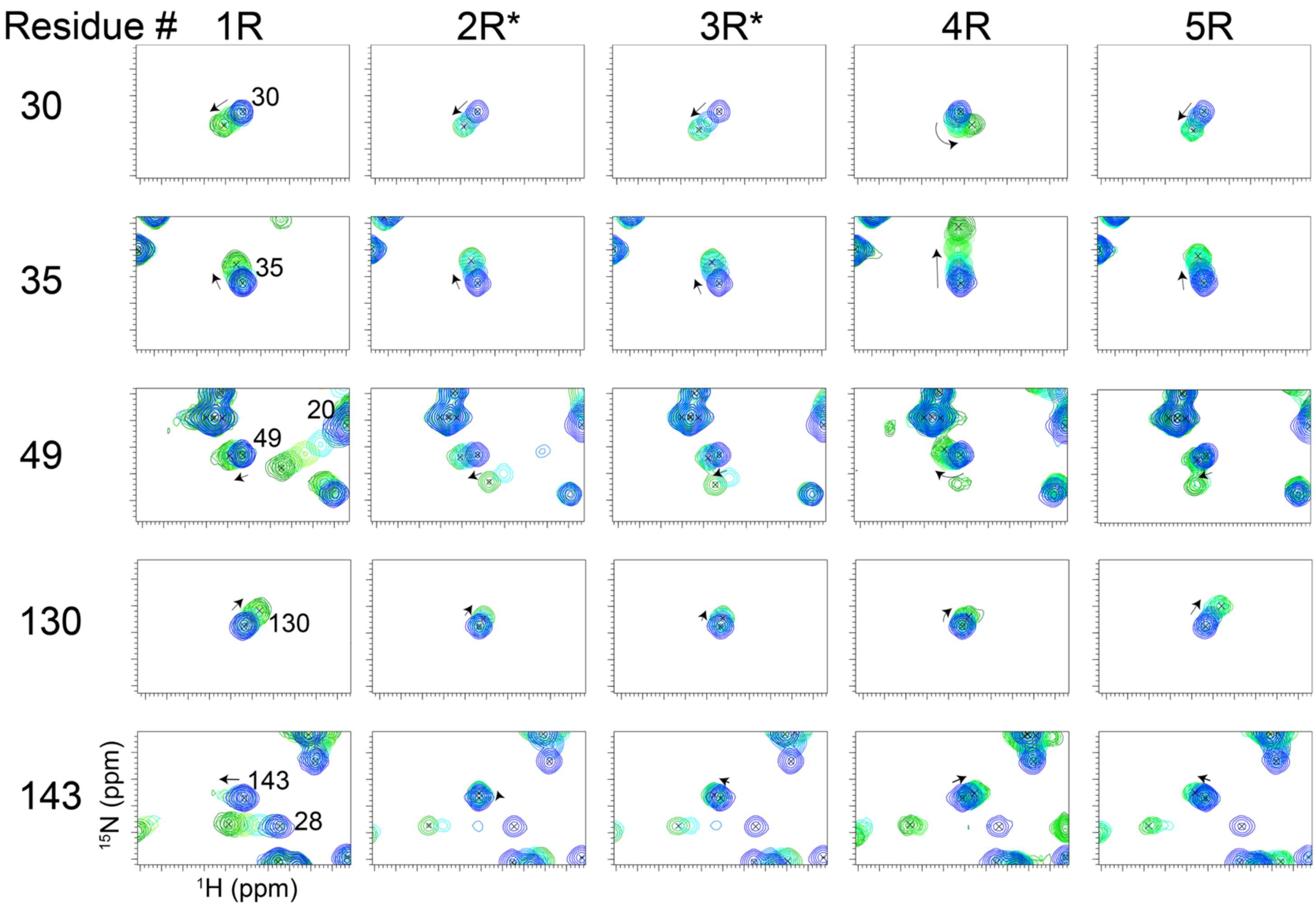

Fig. S9. Residue-by residue chemical shift trajectories as CTD peptides were titrated. (Lower) Linear chemical shift trajectories for marked residues were observed for 1R, 2R, 3R and 5R-CTD titrations. Note that non-linear trajectories, trajectories with different directions, or large CSPs were observed only for 4R-CTD peptide. The 2R and 3R-CTD peptides were titrated up to 2:1 peptide:protein stoichiometric ratio, while 1R-CTD was titrated to 6:1 ratio, and 4R and 5R up to 3:1 ratio. NMR spectra at the start and end of the titration are colored blue and green, respectively. (Upper) Marked residues were mapped onto the Ess1 structure in red.

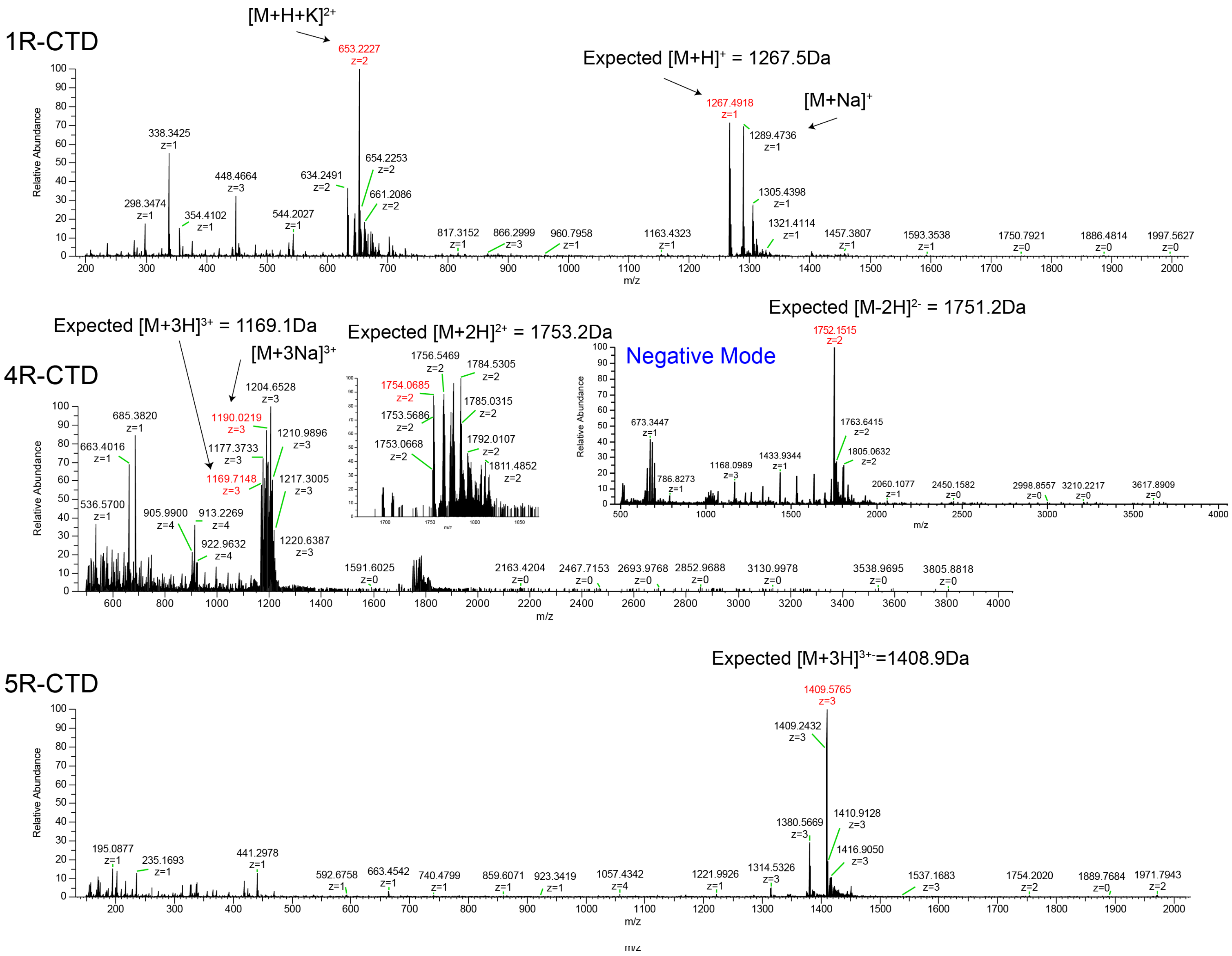

Fig. S10. ESI-MS spectra of CTD peptides. Shown are positive-mode ESI-MS spectra for 1R, 4R and 5R CTD peptides that were used in NMR titration experiments. For 4R-CTD, we also provide negative-mode ESI spectra.

Supplementary Table 1. Small Angle X-ray Scattering Results for Ess1.

**Sample details**	
Organism	*Saccharomyces cerevisiae*
Source	*E. coli* expressed
UniProt sequence ID	P22696 (R8S polymorphism)
Extinction coefficient [A280, 0.1%(w/v)]	1.064
PSV from chemical composition (cm^3 g^{-1})	0.73
MW from chemical composition (Da)	19,710.01
Loading concentration (mg/mL)	4.78; 3.19; 1.59
**SAXS data-collection parameters**	
Instrument/data processing	Cornell High Energy Synchrotron Source G1 beamline (Ithaca, NY) with dual PILATUS 100K-S detector
Wavelength (Å)	1.244
Beam size (μm)	250 x 500; square beam
Camera length (m)	1.14
q measurement range ($Å^{-1}$)	0.0119 - 0.3387
Absolute scaling method	Comparison with scattering from 1mm pure H_2O
Normalization	To transmitted intensity by beam-stop counter
Monitoring for radiation damage	Sample was oscillated to reduce exposure time
Exposure time	4 successive exposures; 1 second each
Sample configuration	30 μL in a sheath-flow cell
Sample temperature (°C)	4
**Software employed for the SAXS data reduction, analysis and interpretation**	
SAXS data reduction	solvent subtraction using PRIMUSqt
Extinction coefficient estimate	protparam (EXPASY)
Calculation of delta-rho and PSV values	hydropro
Basic analyses: Guinier, P(r), V_p	PRIMUSqt from ATSAS 3.0.2
Shape/bead modelling	DAMMIF and DAMMIN from ATSAS 3.0.2
Atomic structure modelling	CRYSOL from PRIMUSqt in ATSAS 3.0.2
Three-dimensional graphic model representations	Chimera

**Structural parameters**			
	Ess1<br>4.78 mg/mL	Ess1<br>3.19 mg/mL	Ess1<br>1.59 mg/mL
Guinier analysis			
I(0) (cm^{-1})	4.39	3.13	1.34
R_g (Å)	21.38	20.83	20.42
q_{min} ($Å^{-1}$)	0.25	0.25	0.36
qR_g max	1.24	1.25	1.34
Coefficient of correlation, R^2	0.99	0.98	0.96
MW from I(0) (Da) (ratio to predicted)	23,400 (1.19)	18600 (0.943)	18900 (0.958)
P(r) analysis			
I(0) (cm^{-1})	4.39 +/- 0.04	3.15 +/- 0.03	1.35 +/- 0.03
R_g (Å)	21.59	21.40	20.95
d_{max} (Å)	69.0	71.0	71.0
q range ($Å^{-1}$)	0.012 - 0.339	0.012 - 0.339	0.012 - 0.339
χ^2 (total estimate from RAW)	0.121	0.115	0.111
MW from I(0) (ratio to predicted)	23400 (1.19)	18600 (0.94)	18900 (0.96)
Porod volume ($Å^{-3}$) (ratio V_p/calculated M)	32,190	28,276	26,999
**Shape model-fitting results**			
DAMMIF<br>*(default parameters, 10 calculations)*			
q range for fitting ($Å^{-1}$)	0 - 0.338	0 - 0.338	0 - 0.338
Symmetry, anisotropy assumptions	P1, none	P1, none	P1, none
NSD (standard deviation)	0.554 (0.017)	0.601 (0.033)	0.601 (0.023)
χ^2 range	0.127 – 0.129	0.115 – 0.116	0.111
Constant adjustment to intensities	Skipped<br>(negative value)	0.0229	0.01
Resolution (from SASRES) (ang)	29 +/- 2	30 +/- 2	29 +/- 2
M estimate as 0.5 x volume of models (Da) (ratio to expected)	19,810 (1.00)	17,595 (0.893)	17,610 (0.893)
DAMMIN (default parameters)			
q range for fitting ($Å^{-1}$)	0 - 0.338	0 - 0.339	0 - 0.338
Symmetry, anisotropy assumptions	P1, none	P1, none	P1, none
χ^2	0.120	0.115	0.111
Constant adjustment to intensities	Skipped<br>(negative value)	0.0256	0.012

**Atomistic modelling**			
	**ScEss1**	**CaEss1**	**HsPin1**
Crystal structures	This study	PDB entry 1YW5	PDB entry 1PIN
q range for all modelling	0 - 0.339	0 - 0.339	0 - 0.339
CRYSOL (with default parameters)			
No constant subtraction			
χ^2	0.209	0.959	1.467
Predicted R_g (Å)	20.69	18.72	17.92
Vol (Å), Ra (Å), Dro (e $Å^{-3}$)	22670, 1.4, 0.075	25492, 1.76, 0.075	23599, 1.4, 0.075
Constant subtraction allowed			
χ^2	0.205	0.942	1.456
Predicted Rg (Å)	20.69	18.76	17.92
Vol (Å), Ra (Å), Dro (e $Å^{-3}$)	23126, 1.8, 0.075	25124, 1.4, 0.075	23599, 1.4, 0.075

Supplementary Table 2. Rotational diffusion tensor characteristics of Ess1.

Diffusion tensor [a]									
	D_{xx} [b]	D_{yy}	D_{zz}	α [c]	β	γ	τ_c (ns)[d]	Anisotropy [e]	Rhombicity [e]
WW domain	0.95 (0.14)	1.11 (0.13)	1.76 (0.20)	145 (11)	117 (9)	156 (27)	13.08 (0.95)	1.71 (0.25)	0.308 (0.067)
PPIase domain	1.01 (0.08)	1.08 (0.07)	1.59 (0.12)	159 (9)	116 (8)	171 (41)	13.60 (0.59)	1.52 (0.14)	0.214 (0.034)
Full-length	1.00 (0.05)	1.09 (0.06)	1.61 (0.10)	159 (6)	115 (5)	172 (30)	13.48 (0.47)	1.54 (0.11)	0.215 (0.026)

[a] Rotational diffusion tensors were determined for either full-length Ess1 or the individual domains in Ess1 by selecting the subset of domain-specific residues.
[b] Principal values of the fully anisotropic diffusion tensor, in 10^7 s^{-1}. Errors (in parentheses) were estimated using 1000 Monte Carlo trials.
[c] Euler angles (in degrees) according to the y-convention characterize the principal axes frame of the diffusion tensor with respect to the PDB coordinate frame of Ess1.
[d] Overall rotational correlation time.
[e] Calculated as defined in (Fushman *et al*., 2004, ref. 18).

Supplementary Table 3. Ess1 binding dissociation constants (K_d) for 1R, 4R and 5R-CTD peptides determined by NMR titrations. Assuming 1 to 1 binding, individual K_ds were determined by following residues with CSP >0.03 ppm at the final titration point (6:1 peptide:protein stoichiometric ratio for 1R-CTD, and 3:1 for 4R and 5R-CTD peptides).

	K_d (μM)				K_d (μM)		
**Residue**	**1R**	**4R**	**5R**	**Residue**	**1R**	**4R**	**5R**
16	119.2	6.9	3.8	84	119.4	2.2	4.5
17	111.1	3.1	2.1	87	180.8		20.6
18	151.9	20.8	7.2	102			9.3
19	153.7	24.3	4.4	103		230.7	
23	148.5	140.1	24.0	109			6.8
25	124.8	24.0	11.1	112		31.8	14.7
26	196.0			113			23.2
27	128.9	4.2	6.9	115		66.8	32.2
28	161.3	10.8	9.8	123	227.8	236.3	35.8
29	141.4	2.3	3.6	127		8.7	3.8
30	103.1		1.6	130	175.2	11.4	7.4
34	233.3	51.3	6.8	131	207.7		
35	274.5	244.7	16.0	133	166.8		
37	153.0	24.5	6.4	134			2.8
38	102.8			135		9.4	10.5
39	153.9	25.1		136			16.5
40	157.5	6.1	4.4	137	128.3		4.1
47			1.8	145	205.8		1.8
48	156.8			146	235.1		
49	216.2			149	100.8		1.7
52	132.6	2.2	1.8	150	113.4		1.7
54			4.4	157	151.8		
58	96.2		1.7	158	122.1		
59	156.4	21.7	3.0	159	176.3		15.1
64	134.1	3.2	8.3	162	71.8		11.8
70	111.0			164	188.7		
73	89.4			168		48.4	
83		2.9					
