# Supplementary material for: Structure analysis suggests Ess1 isomerizes the carboxy-terminal domain of RNA polymerase II via a bivalent anchoring mechanism
Source: Commun Biol. 2021 Mar 25;4:398. doi: 10.1038/s42003-021-01906-8 (PMC7994582; doi:10.1038/s42003-021-01906-8)
Supplement: Supplementary file 3 — Description of Additional Supplementary Files [file 42003_2021_1906_MOESM3_ESM.pdf]

Description of Additional Supplementary Files

File Name: Supplementary Movie 1

Description: Movie showing Ess1 interacting with a 5-repeat CTD peptide, highlighting its ability to remain bound to the WW domain (left) and while engaging the Ess1 PPIase domain (right) to catalyze *cis*/*trans* proline isomerization. The movie is a model for bivalent interaction and catalytic function based on binding and structural studies. The 5R-CTD peptide (green) is shown in ribbon representation with N- terminus on the left and the C-terminus on the right. The pSer5-Pro6 bond being isomerized at the C-terminus is highlighted (purple). The space filling model of Ess1 is color-coded as follows: orange indicates chemical shift perturbations (CSPs) > 0.03 ppm, red are CSPs > 0.1 ppm, and blue are amide resonances that were broadened beyond detection. See text for details.

File Name: Supplementary Data 1

Description: All source data provided as Excel files within a ZIP file called "RAW data sets": Fig 2B source data Fig 2C, 2D TALOS hetNOE Fig 3 source data Fig 4 source data Fig 5A CSPs Fib 5B titration curves
